# Prevalence and Predictors of Peripheral Vascular Disease Amongst Predialysis Hypertensive Chronic Kidney Disease Patients in Southern Nigeria

**DOI:** 10.7759/cureus.36752

**Published:** 2023-03-27

**Authors:** Henry Ovwasa, Henry O Aiwuyo, ‪Ogochukwu Okoye CA, Ejiroghene M Umuerri, Austine Obasohan, Evelyn Unuigbe, Nilum Rajora

**Affiliations:** 1 Family Medicine, Milk River Health Center, Tomiko, CAN; 2 Internal Medicine, Brookdale University Hospital Medical Center, Brooklyn, USA; 3 Nephrology Division, Department of Internal Medicine, Delta State University Teaching Hospital, Oghara, NGA; 4 Medicine, Delta State University, Abraka, NGA; 5 Internal Medicine, Cardiology, Delta State University Teaching Hospital, Oghara, NGA; 6 Department of Medicine, College of Medical Sciences, University of Benin Teaching Hospital, Benin City, NGA; 7 Department of Nephrology, Department of Internal Medicine, University of Benin Teaching Hospital, Benin City, NGA; 8 Department of Internal Medicine, University of Southwestern Medical Center, Dallas, USA

**Keywords:** hypertension, predictors, prevalence, chronic kidney disease, peripheral vascular disease

## Abstract

Background: Peripheral vascular disease (PVD) is an atherosclerotic disease associated with increased morbidity and mortality among chronic kidney disease (CKD) patients. However, despite the substantial burden of PVD in CKD, local data are lacking.

Objective: To determine the prevalence and predictors of PVD in predialysis CKD patients.

Method and Materials: The study was cross-sectional. One hundred fifty hypertensive CKD patients and age- and sex-matched hypertensive non-CKD subjects were consecutively enrolled at the renal unit of Delta State University Teaching Hospital (DELSUTH), Oghara. Structured questionnaires were used to obtain information on participants' demographic data and health status. PVD was defined by an ankle-brachial index of < 0.9 or > 1.4 in either lower extremity. eGFR was calculated from serum creatinine using the MDRD equation.

Results: The mean ages of the study and control groups were 48±14 and 51±15years, respectively. The sex ratio was 3:2 in favour of males for both the study and control groups. The majority of the study group was in CKD stage 4 (44%). The prevalence of PVD was higher among the CKD group compared with controls (24.0% vs. 14.7%). Of the CKD patients with PVD, 11.1% were symptomatic. Predictors of PVD in the study group were eGFR (B=0.010, 95%CI: 0.007-0.013), diastolic BP (B=-0.005, 95%CI: -0.007- -0.002), MAP (B=-0.018, 95%CI: -0.027- -0.008), urinary ACR (B=-0.0036, 95%CI: -0.040- -0.024) and smoking history (p<0.001, OR=14.71).

Conclusion and Recommendation: PVD is common and largely asymptomatic in CKD patients. The predictors of PVD in this study were eGFR, diastolic BP, mean arterial pressure (MAP), urinary albumin to creatinine ratio (ACR), and smoking. A proactive assessment of PVD and early intervention in CKD patients is needed.

## Introduction

The global burden of chronic kidney disease (CKD) has assumed epidemic proportions worldwide in the last decade, and this has been associated with a considerable increase in morbidity and mortality [1,2]. Data from a population-based Southeast Nigerian study documented an overall age and sex-adjusted prevalence of CKD of 11.4% [3]. Cardiovascular diseases, including peripheral vascular disease (PVD), are responsible for more than 50% of deaths in the CKD population [4,5]. 

Peripheral vascular disease (PVD) is an atherosclerotic disease associated with increased morbidity and mortality among chronic kidney disease (CKD) patients [6,7]. CKD is an independent risk factor for the development of PVD, with the risk increasing with worsening renal function [8]. Apart from the traditional risk factors of atherosclerosis in the general population, CKD is associated with unique hemodynamic and metabolic abnormalities such as chronic inflammation, anemia, albuminuria, hypoalbuminemia, and a pro-calcific state [9]. The prevalence of PVD is dependent on several factors, particularly the method of diagnosis, age, gender, and estimated glomerular filtration rate (eGFR) of the population studied [10,11].

Conversely, albuminuria, which is a surrogate for generalized endothelial dysfunction and an independent cardiovascular risk factor, is strongly associated with medial arterial calcification (MAC), which is responsible for higher-than-normal or false-normal ABI in CKD patients [12]. Ankle-brachial indexes (ABI) are a strong marker of cardiovascular diseases (CVD) and are predictive of cardiovascular events and mortality [13].

The burden of PVD in selected populations, such as hypertensive and diabetic patients, has been well-studied in Nigeria [14,15]. However, despite the high morbidity and mortality associated with PVD in CKD patients, there is a paucity of local data. Agaba et al., in a study of type 2 diabetics with end-stage renal disease attending the Jos University Teaching Hospital, reported a prevalence of PAD of 51.7% [16]. The prevalence rate reported by Agaba was much higher than the 24% prevalence rate found in the National Health and Nutrition Examination Survey (NHANES [1999-2000]), which studied subjects with CKD stage 3 or greater [17]. The marked disparity in findings in both studies is perhaps partly due to differences in the degree of renal impairment and a consequence of co-existing diabetes mellitus in one of the studies.

Ankle-Brachial Index (ABI) is the most cost-effective and risk-free atherosclerosis tool in clinical settings. The ankle-brachial index is an objective, simple, reliable, and non-invasive method of diagnosing PVD. Its simplicity, non-invasiveness, and affordability make ABI an easily performed procedure in the office or consulting room [18]. The ankle-brachial index is 95% sensitive and 99% specific for PVD in the general population compared to the angiogram, which is the gold standard for the diagnosis of PVD [19]. In addition, the angiogram is an invasive procedure associated with complications such as allergy to contrast medium, thromboembolism, and contrast-induced nephropathy in general and CKD populations [20]. The main drawback of the ankle-brachial index in the CKD population is its low sensitivity due to artificially normal or higher values, which are thought to reflect the prevalence of medial arterial calcification [21]. Therefore, the PVD prevalence rates earlier reported are likely significantly lower than the actual burden of the condition. Ideally, patients with high suspicion of PVD despite normal or high ABI should be followed up with exercise ABI, toe-brachial index (TBI), or duplex ultrasonography. The 2012 KDIGO guidelines recommend an earlier use of the ABI test in evaluating individuals with pre-dialysis CKD and consideration as candidates for prescription of evidence-based therapies [22]. The classic symptom of intermittent claudication in PVD is often unreliable because it is infrequently present in less than 10%-15% of ABI-diagnosed PVD [23].

Similarly, in the Chronic Renal Insufficiency Cohort (CRIC) study involving stage 3 or greater CKD patients, 15% of participants had an ABI < 0.9, with only 7% of those with ABI-defined PVD presenting with a classical history of intermittent claudication [24]. Among CKD patients, PVD is significantly associated with the male sex, older age, diabetes, smoking, and degree of renal dysfunction [25]. In a Taiwanese study, CKD stage, systolic BP, diastolic BP, and increased pulse pressure were found to positively correlate with PVD prevalence [26]. The objectives of this study are, therefore, to determine the prevalence of PVD in CKD and identify the predictors of PVD among CKD patients.

## Materials and methods

The study was hospital-based descriptive cross-sectional. A total of 150 hypertensive CKD patients as well as age- and sex-matched hypertensive non-CKD subjects, were consecutively enrolled at the renal and cardiology units of Delta State University Teaching Hospital (DELSUTH), Oghara. DELSUTH is a 250-bed state-owned tertiary hospital in Delta State, South-Southern Nigeria. The hospital serves as the main referral center in Delta State and the neighboring states of Edo, Bayelsa, Anambra, and Ondo. Interviewer-administered structured questionnaires were used to obtain information on the demographic data and health status of participants, such as duration of hypertension, history of diabetes mellitus, history of CKD, and smoking. The brachial and ankle systolic pressures were obtained using the automated oscillometric method with Omron M3 Intellisense after having the participant rest for at least 10 minutes. An automated sphygmomanometer cuff of appropriate size (about 12.5cm wide) was applied evenly on the bare arm, with the lower edge at 2.5cm above the antecubital fossa. A cuff about 15cm wide was used for all obese participants. Readings were taken in a supine position on both arms (the higher of the two arms was the reference pressure). The brachial artery was palpated and located. Doppler gel was applied to the skin over the localized brachial artery, and the Doppler probe was held at about 50 degrees to the artery to enhance the quality of the signal obtained [27]. The ABI was then calculated as the quotient of the ankle pressure and the higher of the two arm pressures. PVD was diagnosed by either a history of calf pain and/or intermittent claudication and an ABI <0.9 or >1.4 in either or both legs [28]. Anthropometric measurements of weight, height, and body mass index were obtained.

Blood samples were obtained for the fasting serum lipid profile, fasting blood sugar, and serum creatinine. Urine samples were obtained for the urinary albumin/creatinine ratio (UACR). Estimated GFR was calculated from serum creatinine using the Modification of Diet in Renal Disease (MDRD) equation [29]. CKD was defined and classified according to the 2012 KDIGO guidelines [7]. Diabetic nephropathy was defined by the history of DM, the presence of diabetic retinopathy on fundoscopy, proteinuria > 300mg/g and normal-sized or enlarged kidneys on ultrasonography [7]. Hypertensive nephrosclerosis was defined by a long history of hypertension in a subject older than 45 years, signs of long-standing hypertension, minimal proteinuria/haematuria, and bilaterally shrunken kidneys [7]. Obesity was defined as a BMI equal to or greater than 30 Kg/m2 [30]. Chronic glomerulonephritis was defined by a history suggestive of renal impairment in a subject aged 45 years or less, the absence of signs of longstanding hypertension, proteinuria, haematuria, and bilaterally shrunken kidneys on renal ultrasonography [31]. Hypertension was defined as systolic blood pressure equal to or greater than 140mmHg and/or diastolic blood pressure equal to or greater than 90mmHg [32]. Diabetes mellitus was defined as a self-report of diagnosis by a doctor or other health personnel or a history of use of anti-diabetic agents, elevated fasting blood glucose greater than or equal to 126mg/dl, or random blood glucose greater than or equal to 200mg/dl with symptoms of hyperglycemia [33].

Data analysis

IBM Corp. Released 2013. IBM SPSS Statistics for Windows, Version 22.0. Armonk, NY: IBM Corp. was used for data management and analysis. Continuous variables were expressed as the mean (SD) for unskewed data or the interquartile range for skewed data. Differences in continuous and categorical variables between the CKD and non-CKD groups were assessed by the student's t-test and chi-square analysis, respectively. A p-value of <0.05 was considered statistically significant.

Predictors and associations were determined by multiple linear regression and logistic regression analysis, respectively.

## Results

Sociodemographic and health characteristics of the study population

**Figure 1:**
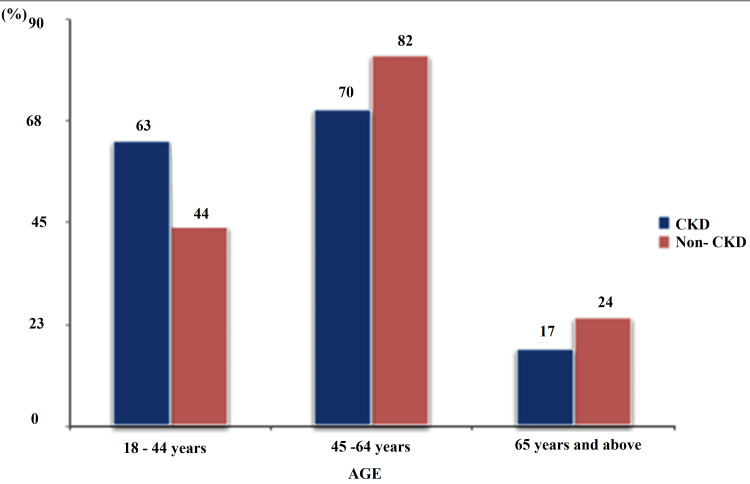
Age distribution amongst study population X^2^(p-value)= 5.52(0.063)

One hundred and fifty hypertensive CKD subjects and one hundred and fifty hypertensive non-CKD comparative participants who fulfilled the inclusion criteria were recruited. The mean ages of the study and comparative groups were 48.6113.93 years and 51.015.45 years, respectively (p-value 0.186). Eighty-nine percent of the hypertensive CKD group and 84% of the hypertensive non-CKD comparative group were younger than 65 years of age. Forty-six-point-seven percent of the study group and 54.7% of the comparative group were in the 45-64 years age group. There was no significant difference in the age distribution between the study and the comparative group (x2=5.52, p=0.063) (Figure 1). There was an equal number of males and females among the study group and comparative group (92 males and 58 females), making a male-to-female ratio of 3:2. Among the CKD group, CKD stages 2, 3, 4, and 5 prevalence was (24) 16%, (45) 30%, (66) 44%, and (15) 10%, respectively. One hundred and eight (72%) of the study group had no history of smoking, 30 (20%) had a previous history of smoking, and only 12 (8%) had a current history of smoking. Of the CKD patients with PVD, only four (11.1%) were symptomatic.

Clinical and biochemical characteristics of the study population

The CKD group had significantly lower mean body mass index (BMI), packed cell volume (PCV), serum calcium, and albumin compared to the non-CKD group.

On the other hand, systolic blood pressure (SBP), diastolic blood pressure (DBP), mean arterial pressure (MAP), pulse pressure, serum phosphate, serum calcium-phosphate product, UACR, and serum creatinine were significantly higher among the hypertensive CKD group than the hypertensive non-CKD group.

There was no significant difference in the mean age and ankle-brachial index between the CKD group and the comparative group (Table 1).

**Table 1:** Clinical and biochemical characteristics of respondents *significant at p<0.05; **presented as the median (IQR) and compared using the Mann-Whitney U test BMI: body mass index, DBP: diastolic blood pressure, ABI: ankle brachial index, PP: pulse pressure, MAP: mean arterial pressure, PCV: packed cell volume, Ca/P: calcium phosphate product, UACR: urinary albumin creatinine ratio.

	CKD N=150 Mean (SD)	NON-CKD N=150 (SD)	t	P
Age (years)	49.83(12.85)	51.04(15.45)	-1.34	0.186
BMI (Kg/m^2^)	27.43(4.61)	31.56(5.21)	-7.29	<0.001*
SBP (mmHg)	170.10(24.12)	153.90(23.63)	5.15	<0.001*
DBP (mmHg)	91.35(9.71)	86.46(9.43)	4.42	<0.001*
ABI	0.96(0.09)	0.95(0.04)	0.88	0.382
PP (mmHg)	75.82(18.80)	67.44(21.14)	1.90	<0.001*
MAP (mmHg)	116.15(14.88)	108.94(12.08)	4.61	<0.001*
PCV (%)	35.25(8.24)	40.87(4.49)	-7.34	<0.001*
Serum calcium (mg/dl)	7.11(2.02)	9.15(0.57)	-11.91	<0.001*
Serum phosphate (mg/dl)	7.16(1.36)	4.22(0.27)	25.98	<0.001*
Serum Ca/P	56.75(9.95)	38.60(3.59)	21.02	<0.001*
Serum albumin (mg/dl)	3.62(0.88)	4.13(0.96)	-1.92	<0.001*
Serum creatinine (mg/dl)	2.26(1.04)	1.07(0.20)	13.78	<0.001*
UACR (mg/g)**	100.50(456.50)	22.00(241.0)	6460.00	<0.001*

**Figure 2:**
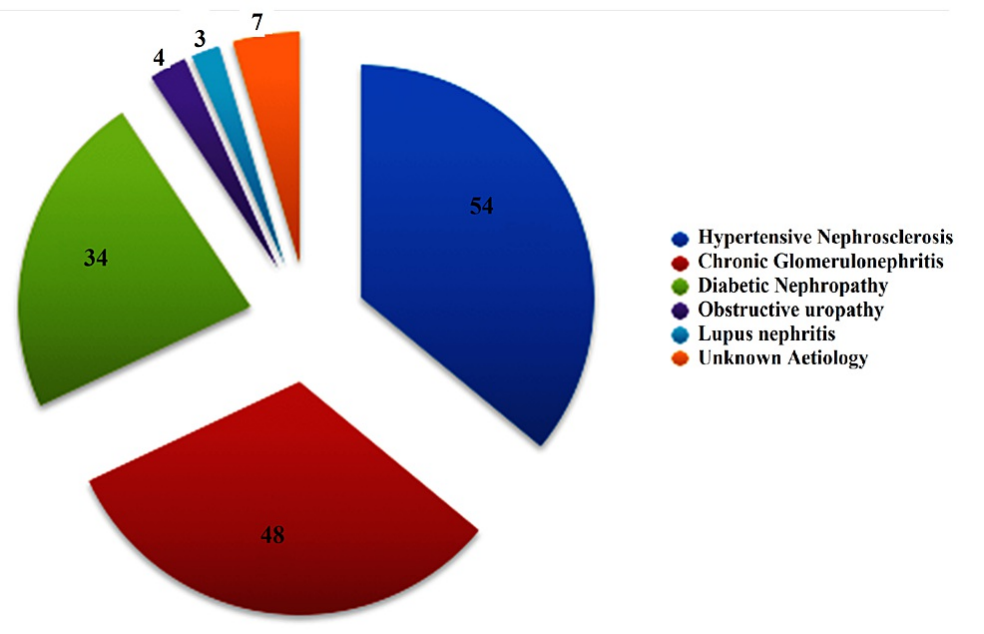
Distribution of aetiology of CKD amongst cases BMI: body mass index, DBP: diastolic blood pressure, ABI: ankle brachial index, PP: pulse pressure, MAP: mean arterial pressure, PCV: packed cell volume, Ca/P: calcium phosphate product, UACR: urinary albumin creatinine ratio.

The most common causes of CKD were hypertensive nephrosclerosis (36%), chronic glomerulonephritis (32%), and diabetic nephropathy (22.6%) (Figure 2).

Prevalence of PVD amongst CKD and non-CKD groups

Peripheral vascular disease was significantly more prevalent in the hypertensive CKD group than the hypertensive non-CKD group (p-value=0.04, OR=1.84, CI= 1.68-3.45) (Table 2).

**Table 2:** Prevalence of PVD amongst CKD and non-CKD groups

Peripheral Vascular Disease	Chronic Kidney Disease Frequency (%)		Total
	Present	Absent	
Yes	36 (24% )	22 (14.7%)	58
No	114	128	242
	OR = 1.84; CI: 1.68 - 3.45; p = 0.04;X^2^=5.61		

**Figure 3:**
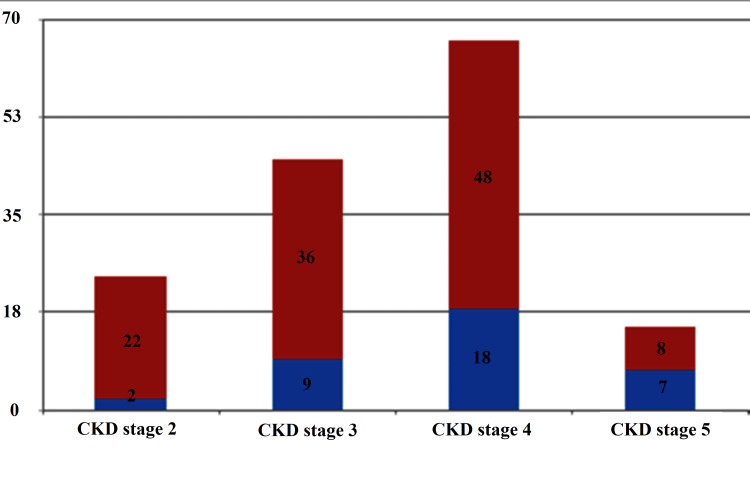
Prevalence of PVD and stages of CKD PVD: peripheral vascular disease; Chi-square = 10.848; p-value = 0.013

In this study, the prevalence of PVD generally increased with deteriorating renal functions, being lowest in CKD stage 2 and highest in CKD stage 5 at 8% and 47%, respectively, p = 0.013 (Figure 3).

Ankle Brachial Index is strongly and positively correlated with eGFR, PCV, serum phosphate, calcium-phosphate product, and urine albumin-creatinine ratio but negatively correlated with SBP, DBP, MAP, and serum LDL (Table 3). 

**Table 3:** Correlation between cardiovascular risk indices and ankle-brachial index amongst CKD patients ABI: ankle brachial index, eGFR: estimated glomerular filtration rate, SBP: systolic blood pressure, DBP: diastolic blood pressure, PP: pulse pressure, MAP: mean arterial pressure, PCV: packed cell volume, Ca/P: calcium phosphate product, TC: total cholesterol, TG: triglyceride, LDL: low density lipoprotein, HDL: high density lipoprotein, Urine-ACR: urinary albumin creatinine ratio

CKD Group n = 150	r	(p-value)
eGFR	0.63	(<0.001)
Age	-0.108	(0.069)
SBP	-0.12	(0.039)
DBP	-0.40	(<0.001)
PP	0.08	(0.175)
MAP	-0.25	(<0.001)
PCV	0.47	(<0.001)
Calcium	-0.08	(0.160)
Phosphate	0.54	(<0.001)
Ca/P	0.51	(<0.001)
TC	0.01	(0.912)
TG	0.09	(0.136)
LDL	-0.19	(0.001)
HDL	-0.09	(0.132)
Urine-ACR	0.26	(<0.001)

When cardiovascular risk indices were subjected to linear regression analysis against ABI, eGFR, DBP, MAP, serum phosphate, and urinary ACR were found to be statistically significant and are, therefore, independent predictors of PVD, as shown in Table 4.

**Table 4:** Linear regression analysis of cardiovascular risk indices vs. ABI Dependent Variable: ABI, significant at p-value <0.05, PCV: packed cell volume

	Unstandardized Coefficients				Standardized Coefficients		t		Sig.			95% Confidence Interval for B		
	B		Std. Error		Beta							Lower Bound	Upper Bound	
(Constant)		2.304		.500				4.609		.000	1.320			3.288
eGFR		.010		.002		.412		6.192		.000*	.007			.013
Systolic BP		-.005		.003		-.120		-1.696		.091	-.010			.001
Diastolic BP		-.005		.001		-.168		-3.699		.000*	-.007			-.002
Mean Arterial Pressure		-.018		.005		-.258		-3.541		.000*	-.027			-.008
PCV		.039		.006		.290		5.950		.859	-.051			-.026
Serum Phosphate		-.007		.041		-.014		-1.177		.015*	-.074			-.008
Ca-P Product		.002		.006		.026		.372		.710	-.009			.013
Low density Lipoprotein		-.003		.001		-.155		-3.886		.653	-.005			-.002
urine albumin/creatinine ratio		-0.036		.000		-.020		-.450		.000*	-.040			-.024

## Discussion

Cardiovascular disease (CVD) is the leading cause of morbidity and mortality in patients at every stage of chronic kidney disease [1,2]. The incremental risk of CVD in those with CKD compared to the age- and sex-matched general population ranges from 10-200 fold, depending on the stage of CKD. 4 Most patients with CKD succumb to CVD before ever reaching end-stage renal disease (ESRD) [5].

Peripheral vascular disease is an indicator of systemic atherosclerosis, and CKD is a recognized risk factor for atherosclerosis due to the increased prevalence of both traditional and non-traditional risk factors of atherosclerosis among the CKD population. The prevalence of PVD is dependent on several factors, particularly the method of diagnosis, age as well as the gender of the population studied.

In this study, the mean ages of the CKD and non-CKD groups were 49.61 ± 13.93 years and 51.04 ± 15.45 years, respectively, which are comparable with earlier studies that reported the mean ages of CKD patients as being between the 3rd and 5th decades [3]. This, however, contrasts with a report from western countries in which over 50% of the CKD population is aged 65 and above [17]. Thus, CKD and its attendant increased morbidity and mortality carry a higher economic burden in Sub-Saharan Africa, including Nigeria, than in developed countries.

Fifty-nine point three percent (59.3%) of the CKD group in this study presented at CKD stage 4; only 13.3% were at CKD stage 2. This confirms an earlier report by Ulasi et al. in a south-east Nigerian study, in which the majority of the patients studied presented in CKD stages 4 and 5 [3]. This finding implies that CKD patients present late to the nephrologists in most developing countries, where complications of CKD, including cardiovascular diseases, are already advanced and largely irreversible.

The prevalence of PVD (defined as an ABI less than 0.9 or greater than 1.4) of 24% in the hypertensive CKD group was significantly higher than in the comparative group, as was expected. The finding in this study is consistent with that described in the NHANES study but higher than the 15% prevalence in the Chronic Renal Insufficiency Cohort (CRIC) study involving 3612 predominantly stage 3 CKD patients [17,24]. The difference in prevalence may be due to disparities in the characteristics of the study populations, such as age, gender, smoking history, and CKD stage. In our study, no participant had an ABI > 1.4. The ankle-brachial index is a safe and convenient tool commonly used as a surrogate marker for PVD, compared with gold-standard angiography [19]. The finding of intermittent claudication being present in only 11.1% of ABI-diagnosed PVD is similar to the observation by McDermott et al., who reported intermittent claudication in 10%-15% of PVD [23]. Thus, a history of intermittent claudication alone is not reliable in diagnosing PVD. The study also demonstrated that the prevalence rate of PVD increases with decreasing eGFR. This may be due to the increased prevalence of both traditional and non-traditional atherosclerosis risk factors with increasing CKD stages. Recognized risk factors for PVD include male gender, older age, diabetes, smoking, hypertension, and dyslipidemia. In our study, CKD stages, hypertension, albuminuria, serum phosphate, and smoking were independent predictors of PVD. Webb et al. reported that older age, smoking, hypertension, and hypertriglyceridemia are associated with PVD [9]. Smoking is a strong modifiable traditional risk factor for the development of atherosclerosis and is known to increase the prevalence of coronary artery disease, PVD, and stroke. Peripheral vascular disease is associated with an increased risk of both cardiovascular and all-cause mortality among people with CKD. It is, therefore, pertinent to follow the recommendations of the 2012 KDIGO consensus document on the management of PVD in relation to early identification of PVD and the evaluation of patients with intermittent claudication [22]. The main benefit of timely identification and intervention is a reduction in the risk of myocardial infarction, stroke, and cardiovascular mortality.

This study, despite its strength of being the first on PVD in the CKD population in South-South Nigeria, had limitations, such as the ABI used in the evaluation of PVD is associated with falsely elevated values in CKD. Toe-Brachial Index (TBI), which correlates better with PVD in CKD, was not done due to logistics. Similarly, because it is a single-center cross-sectional study, patient selection might be biased, and our conclusion should not be generalized. However, the findings in the study will not only provide local data but also be a platform for future studies on the subject.

## Conclusions

PVD is common and largely asymptomatic in CKD patients. The burden of CKD alone is worrisome, let alone complications with PVD when they occur. The predictors of PVD in this study were eGFR, diastolic BP, MAP, urinary ACR, and smoking. Therefore, the index study not only strengthens the prevalence of PVD in CKD patients but also advocates a need for proactive assessment of PVD and early intervention in CKD patients.
